# The role of Sphingomyelin synthase 2 (SMS2) in platelet activation and its clinical significance

**DOI:** 10.1186/s12959-021-00282-x

**Published:** 2021-04-28

**Authors:** Yifan Guo, Lin Chang, Ge Zhang, Zhanyan Gao, Hao Lin, Yuting Zhang, Liang Hu, She Chen, Bing Fan, Si Zhang, Ruyi Xue

**Affiliations:** 1 Department of Cardiology, Shanghai Institute of Cardiovascular Diseases, Zhongshan Hospital, Fudan University, 200032 Shanghai, China; 2 Department of Biochemistry and Molecular Biology, NHC Key Laboratory of Glycoconjugates Research, School of Basic Medical Sciences, Fudan University, 200032 Shanghai, China; 3 Shanghai Medical College, Fudan University, 200032 Shanghai, China; 4 Department of Cardiology, the First Affiliated Hospital of Zhengzhou University, Cardiovascular Institute of Zhengzhou University, 450052 Zhengzhou, China; 5 Department of Gastroenterology and Hepatology, Shanghai Institute of Liver Diseases, Zhongshan Hospital, Fudan University, 200032 Shanghai, China

**Keywords:** SMS2, Platelet, Thrombosis, D609

## Abstract

**Background:**

Sphingomyelin (SM) is an essential component of biological lipid rafts, and it plays an indispensable role in maintaining plasma membrane stability and in mediating signal transduction. The ultimate biosynthesis of SM is catalyzed by two sphingomyelin synthases (SMSs) namely SMS1 and SMS2, which are selectively distributed in the trans-Golgi apparatus and the plasma membrane. It has been demonstrated that SMS2 acts as an irreplaceable molecule in the regulation of transmembrane signaling, and loss of SMS2 has been reported to worsen atherosclerosis and liver steatosis. However, the function of SMS2 in platelet activation and its association with the pathological process of thrombosis in acute coronary syndrome (ACS) and portal hypertension (PH) remain unclear.

**Methods:**

In this study, we tested the role of SMS2 in platelet activation and thrombosis using SMS2 knockout (SMS2 –/–) mice and SMS2-specific inhibitor, D609. Furthermore, we detected SMS2 expression in patients with ACS and PH.

**Results:**

SMS2 –/– platelets showed significant reduction in platelet aggregation, spreading, clot retraction and in vivo thrombosis. Similar inhibitory effects on platelet activation were detected in D609-treated wild-type platelets. PLCγ/PI3K/Akt signaling pathway was inhibited in SMS2 –/– platelets and D609-treated wild-type platelets. In addition, we discovered that platelet SMS2 expression was remarkably increased in patients with ACS and PH, compared with healthy subjects.

**Conclusions:**

Our study indicates that SMS2 acts as a positive regulator of platelet activation and thrombosis, and provides a theoretical basis for the potential use of D609 in anti-thrombosis treatment.

**Supplementary Information:**

The online version contains supplementary material available at 10.1186/s12959-021-00282-x.

## Background

Platelet activation plays an important role in a number of thrombotic diseases, especially in the pathological process of acute coronary syndrome (ACS) [1]. Following vessel wall injury, platelets in the bloodstream are instantly stimulated by exposed subendothelial components, and are converted to an active state which enables them to form a thrombus to control bleeding. It is well recognized that over-activated platelets induce excessive thrombosis in response to plaque rupture or erosion of an atherosclerotic coronary artery, resulting in myocardial ischemia [2, 3]. Clinically, dual antiplatelet therapy is fundamental to the prevention of ischemic events in patients with ACS; however, several patients continue to suffer from adverse clinical outcomes after standard dual antiplatelet treatment, which might be due to high residual platelet reactivity [4–6]. Moreover, accumulating evidence indicates that platelet hyperreactivity and thrombosis are important contributors to sinusoidal portal hypertension (PH) and fibrosis progression [7–9]. These effects might be mediated by excessive platelet-derived serotonin, which could stimulate calcium influx into sinusoidal endothelial cells and cause fenestrae contraction, resulting in reduced sinusoidal flow [10–12].

Sphingomyelin (SM) is the most abundant sphingolipid in the cell, and acts as a key modulator in various physiological processes, including cell proliferation, differentiation, apoptosis, oxidative stress, and immune inflammatory responses [13]. The level of SM allows multiple membrane receptors to exert their functionalities through its crucial role in maintaining the stability of the cell membrane and in mediating the transmembrane signaling pathway. A pivotal enzyme involved in the final step of the biosynthetic pathway of SM is sphingomyelin synthase (SMS). Three members of the SMS gene family namely SMS1, SMS2, and SMSr are found in the cell, but only SMS1 and SMS2, which are selectively distributed in the trans-Golgi apparatus and plasma membrane, have catalytic activity [14, 15]. Previous studies have shown that downregulation of SMS2 exerts protective effects against a variety of clinical conditions, such as atherosclerosis [16, 17] and liver steatosis [18, 19]. However, the underlying mechanism of SMS2 involvement in these diseases has not been fully elucidated. Since platelets require an integrated membrane structure to ensure that their membrane receptors function adequately, it is natural to assume that SMS2, as a regulator of platelet signal transduction and activation, might be involved in the pathogenesis of thrombotic diseases such as ACS and PH.

In this study, we demonstrated the expression of SMS2 in platelets and discovered that the level of SMS2 expression was higher in patients with ACS and PH compared with healthy subjects. In addition, we found that SMS2 deficiency in SMS2 knockout (SMS2 –/–) mice reduced platelet activation and thrombosis, leading to a downregulation of the PLCγ/PI3K/Akt pathway. Similar effects were detected when the SMS2-specific inhibitor D609 was used. These results provide a theoretical basis for SMS2 inhibition in the treatment of patients with ACS and PH.

## Methods

### Reagents

SMS2 antibody (ab58074) was obtained from Abcam, while phospho-PLC_γ_2-Tyr1217 (3871), PLC_γ_2 (5690), phospho-PI3 Kinase p85-Tyr458/p55-Tyr199 (17,366), PI3 kinase p85 (4257), phospho- phospho-Akt-Ser473 (4060), and Akt (4691) antibodies were purchased from Cell Signaling Technology. PE-conjugated mouse CD41 (558,040) and FITC-conjugated mouse CD61 (561,911) were purchased from BD PharMingen. PE-conjugated mouse Jon/A (m023-2) was obtained from Emfret Analytics. FITC-conjugated mouse fibrinogen (ab92792) was purchased from Abcam. APC-conjugated human CD62P (12-0626-82) was acquired from Invitrogen. D609 (HY-70,072) was obtained from MedChem Express. Fibrinogen was purchased from Sigma-Aldrich. ADP, collagen, and thrombin were acquired from Chono-Log. FITC-phalloidin (C1033) was purchased from Beyotime. SMS2 –/– mice were provided by Dr. Ding from the College of Pharmacy, Fudan University. Wild-type (WT) mice and rats were purchased from JiesiJie Experimental Animal Company (Shanghai, China).

### Platelet and peripheral blood mononuclear cell preparation

A total of 58 patients were enrolled in this study; 12 patients were diagnosed with stable angina pectoris (SAP), 31 were diagnosed with ACS, and 15 were diagnosed with PH. A total of 27 healthy volunteers matched for age and sex were also included. Platelets and peripheral blood mononuclear cells (PBMCs) obtained from patients and healthy subjects were prepared as previously described [20]. Platelets obtained from whole blood were isolated by two-step centrifugation and re-suspended in modified Tyrode’s buffer [21]. The purity of the washed platelets was checked by routine blood tests using a blood cell counter (BC-2800Vet, Mindray) (purity > 99 %). Experiments involving human subjects were performed in accordance with the Declaration of Helsinki and approved by the institutional review board of Fudan University. Informed consent was obtained from all participants. Animal experiments were conducted according to the criteria described in the “Guide for the Care and Use of Laboratory Animals” published by the National Institutes of Health (NIH publication 86 − 23, revised 1985). For all animal experiments, WT and SMS2 –/– mice were paired with animals of similar age, weight, and sex ratio.

### Western blot

The cells and washed platelets were lysed in radioimmunoprecipitation assay (RIPA) buffer. Cellular debris was discarded by centrifugation at 12,000 × g for 10 min at 4 °C. The supernatant was boiled and subjected to SDS-PAGE, after which it was electro-transferred to a polyvinylidene fluoride (PVDF) membrane. The PVDF membrane was blocked in TBS solution containing 5 % non-fat powdered milk, incubated with specific antibodies, and analyzed by chemiluminescence detection.

### Polymerase chain reaction

Total RNA was extracted using the TRIzol reagent (TaKaRa, Japan). Reverse transcription was used to reverse transcribe 500ng RNA to cDNA. A polymerase chain reaction (PCR) kit (TaKaRa, Japan) was utilized [22]. The sequences of primers used in the PCR reactions include the following: (1) Human/Mouse CD14-UP: 5’-CTTAAAGCGGCTTACGGTGC-3’, (2) Human/Mouse CD14-DN: 5’-CAGCATCCCGCAGTGAATTG-3’, (3) Human/Rat GAPDH-UP: 5’-GGAGCGAGATCCCTCCAAAAT-3’, (4) Human/Rat GAPDH-DN: 5’-GGCTGTTGTCATACTTCTCATGG-3’, (5) Mouse GAPDH-UP: 5’-GCCCAGAACATCATCCCTG-3’, (6) Mouse GAPDH-DN: 5’-TCAGATCCACGACGGACACA-3’, (7) Mouse SMS2-UP: 5’-GTGGCGGACAATGGATATCATAGAGACAGC-3’, (8) Mouse SMS2-DN: 5’-GATAAGGTCTTGGGTTTGCCCTTGCC-3’, (9) Human/rat SMS2-UP: 5’-CTTAGCCCTCCACTCCC-3’, and (10) Human/rat SMS2-DN: 5’-CAGAATCTGCGTCCCAC-3’.

### Platelet flow cytometry analysis

Platelet count was adjusted to 20 × 10^9^/L with modified Tyrode’s buffer. To measure the expression of total and activated integrin αIIbβ3 on the membrane surface of platelets, washed platelets were either left in a resting state or were activated by thrombin (0.05 U/mL). They were then incubated with PE-conjugated mouse CD41, FITC-conjugated mouse CD61, PE-conjugated mouse Jon/A, and FITC-conjugated mouse fibrinogen antibodies at room temperature for 20 min. In addition, PE-conjugated human SMS2 and APC-conjugated human CD62P were used to detect SMS2 expression and 0.05 U/mL thrombin-induced P-selectin exposure on platelet surface membrane from patients with ACS. Data were analyzed using a flow cytometer (BD FACSAria, IIF0893488).

### Platelet aggregation, spreading, and clot retraction

In the platelet aggregation experiment, mouse and human platelets were re-suspended and adjusted to a concentration of 300 × 10^9^/L with modified Tyrode’s buffer. Aggregation in response to thrombin, collagen, ADP, and U46619 at their indicated concentrations was measured using a turbidimetric platelet aggregometer at 37 °C with stirring at 1000 rpm. Platelets were preincubated with aspirin (100 µM) for 20 min prior to ADP-induced aggregation. The procedure has been described previously [20]. Analysis of platelet spreading on immobilized fibrinogen was performed as described by Zhang et al. [23]. Washed platelets (30 × 10^9^/L) were added onto fibrinogen (200 µg/mL)-coated coverslips at 37 °C for different lengths of time, as indicated. The platelets were then fixed, permeabilized, and stained with FITC-conjugated phalloidin at room temperature. Clot retraction was performed under thrombin (0.2 U/mL) stimulation as described previously [24]. The platelet count was adjusted to 500 × 10^9^/L. A photograph of the retracted clot was taken at the indicated time point, and the size of the clot was quantified using ImageJ software.

### FeCl_3_-induced thrombus formation in mouse mesenteric arteriole

Intravital microscopy of FeCl_3_-induced thrombus formation in mouse mesenteric arterioles was conducted as previously described [23, 25]. Platelets (500 × 10^9^/L) labeled with the fluorescent dye Can-AM (1:200) were intravenously injected into WT and SMS2 –/– mice via caudal venipuncture. Thrombosis after FeCl_3_ infiltration was recorded using intravital microscopy. The D609 inhibition group received an intragastric administration of 10 mg/kg D609 prior to anesthesia.

### Arteriovenous shunt thrombosis

The procedure for inducing arteriovenous shunt thrombosis was performed as described previously [26, 27]. D609 and a control solvent (1 mL/min) were injected through the jugular vein. The shunt was maintained for 15 min to allow thrombus formation on the cotton thread. Thrombus weight was determined by subtracting the weight of the dry cotton thread from that of the cotton thread with the thrombus.

### Statistical analysis

Experimental data were analyzed using GraphPad Prism version 7.0 (GraphPad Software, La Jolla, CA, USA). Continuous variables consistent with a normal distribution were compared using parametric tests. Discontinuous variables and data with an abnormal distribution were evaluated using the Wilcoxon rank-sum test. Data are presented as the mean ± standard deviation (SD) calculated from at least three independent experiments. Normality was assessed using the Kolmogorov–Smirnov test. All statistical tests were two-tailed and statistical significance was set at a p-value < 0.05.

## Results

### Expression of SMS2 in human, mouse, and rat platelets

**Fig. 1:**
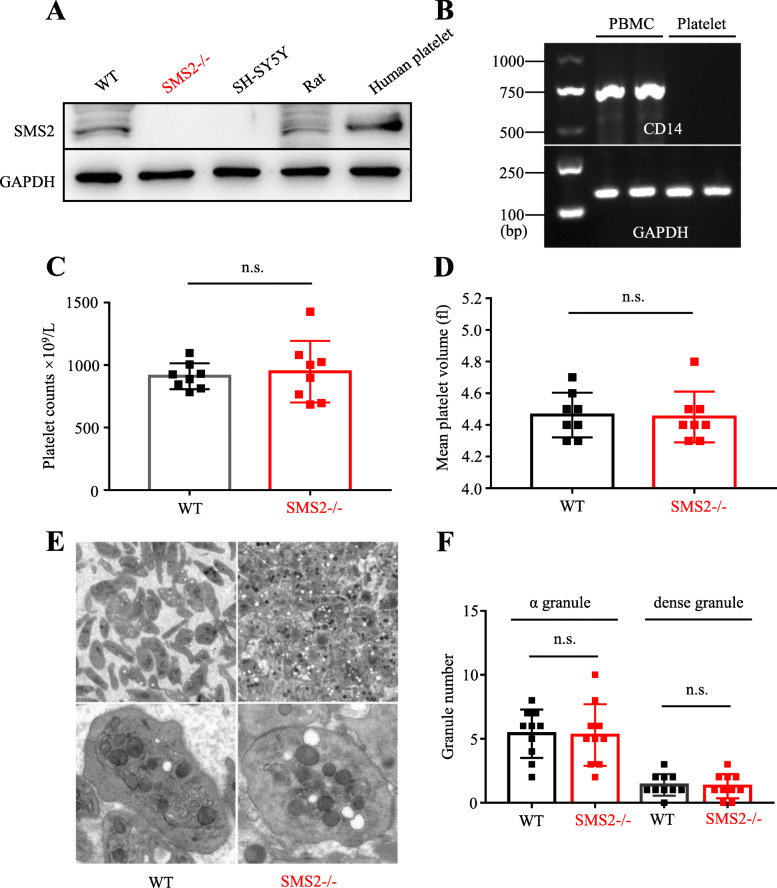
Human, mouse and rat platelets express SMS2. **a** Western blot detection of SMS2 in platelets from WT mice, SMS2 –/– mice, SH-SY5Y cell line, rats and human subjects. **b** PCR detection of monocyte-specific marker CD14. **c**, **d** Platelet count and mean volume in the peripheral blood of WT (black) and SMS2 –/– (red) mice (*n* = 8, n.s. *P* > 0.05). **e**, **f** Morphology of WT and SMS2 –/– platelets observed by transmission electron microscope. The average number of alpha and dense particles were calculated and no significant difference was found between WT and SMS2 –/– platelets (*n* = 10, n.s. *P* > 0.05)

Human, mouse, and rat platelets express SMS2 protein, as detected by western blotting and PCR. The SH-SY5Y cell line was used as a negative control (Fig. 1a, Supplementary Fig. 1A). The PBMC-specific marker CD14 was not detected by PCR performed on the protein samples obtained from platelets, confirming that those samples were not contaminated by PBMCs (Fig. 1b). Blood tests showed that the platelet count (Fig. 1c) and mean platelet volume (Fig. 1d) did not differ significantly between SMS2 –/– and WT platelets. Transmission electron microscopy (TEM) revealed that the morphology of SMS2 –/– platelets changed from an original discoid shape to a more flat and round shape (Fig. 1e), while the number of α-granules and dense granules showed no significant difference between WT and SMS2 –/– platelets (Fig. 1f).

### Reduction in platelet activation due to SMS2 deficiency

**Fig. 2:**
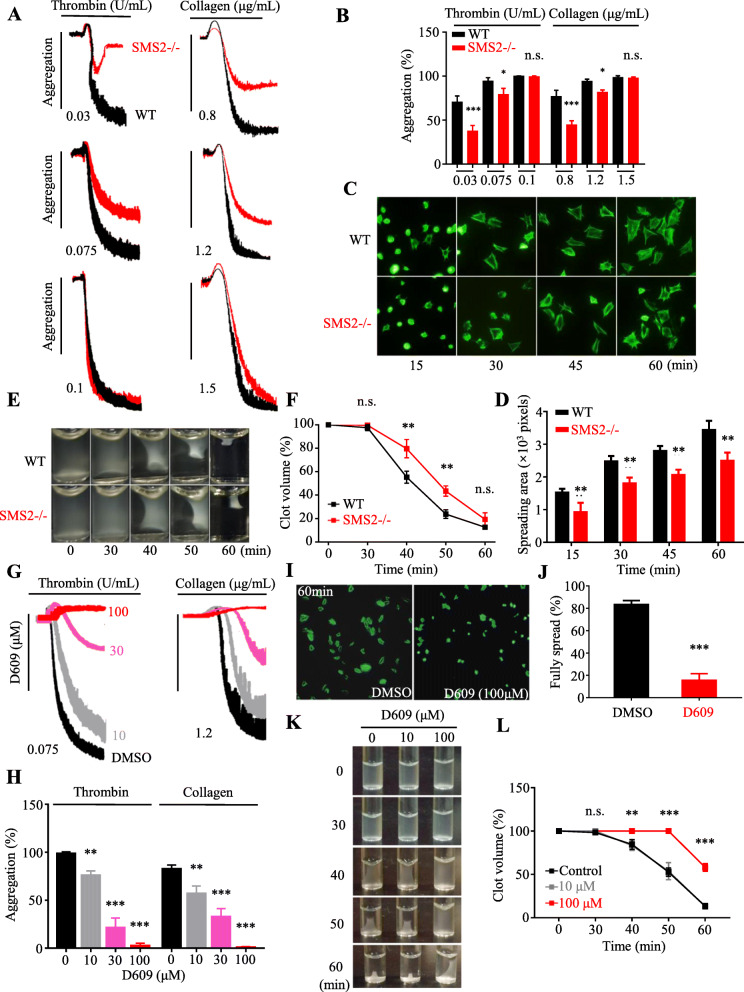
SMS2 deficiency reduced platelet activation in aggregation, spreading and clot retraction experiments. **a**, **b** Platelets from WT and SMS2 –/– mice were re-suspended with Tyrode’s buffer. Thrombin- and collagen-induced aggregation were recorded in a platelet aggregator (black, WT, red, SMS2 –/–, *n* = 4, n.s. *P* > 0.05, * *P* < 0.05, *** *P* < 0.001). **c**, **d** WT and SMS2 –/– platelets spreading on fibrinogen-coated slides. Spreading areas were quantified at indicated time points (*n* = 4, ** *P* < 0.01). **e**, **f** WT and SMS2 –/– platelets were stimulated with thrombin (0.2 U/mL) to coagulate. Images were pictured at indicated timepoints (*n* = 4, ** *P* < 0.01). **g**, **h** Thrombin- and collagen-induced human platelet aggregation by adding D609 (red curve, 100 µM, purple curve, 30 µM, gray curve, 10 µM, black curve, DMSO control, *n* = 4, ** *P* < 0.01, *** *P* < 0.001). **i**, **j** Human platelets spreading on fibrinogen with or without D609 (100 µM). Fully spread ratios were quantified (*n* = 4, *** *P* < 0.001). **k**, **l** Washed platelets added with thrombin (0.2 U/mL) and D609 at indicated concentration to coagulate (red, 100 µM, gray 10 µM, black, DMSO control, *n* = 4, n.s. *P* > 0.05, ** *P* < 0.01, *** *P* < 0.001)

It is well known that platelet signal transduction stimulated by agonists, such as collagen and thrombin, greatly depends on the integrity of lipid rafts [28].Thus, we assumed that SMS2 could act as a regulator of platelet activation by maintaining membrane stability. To test our hypothesis, SMS2 –/– mice were used to show that SMS2 deficiency impaired collagen- and thrombin-induced platelet aggregation. Aggregation induced by thrombin (0.03 U/mL) and collagen (0.8 µg/mL) were lower by 42 and 35 %, respectively, in SMS2 –/– platelets compared to WT platelets. When medium concentrations of thrombin (0.075 U/mL) and collagen (1.2 µg/mL) were used, SMS2 –/– platelets still experienced lower aggregation rates of 22 and 28 %, respectively, compared to WT platelets. However, an increase in the concentrations of thrombin (0.1 U/mL) and collagen (1 µg/mL) led to similar platelet aggregation rates between the two groups (Fig. 2a, b). Similarly, SMS2 –/– platelets displayed decreased aggregation in response to ADP or U46619 stimulation at low and medium concentrations, but had unchanged aggregation at high concentration compared with WT platelets (Supplementary Fig. 2A, B). To further test whether the loss of SMS2 affected platelet outside-in signaling downstream of integrin αIIbβ3 activation, platelet spreading and clot retraction experiments were performed. Compared with WT platelets, spreading of SMS2 –/– platelets on immobilized fibrinogen was significantly hampered at 15, 30, 45, and 60 min (Fig. 2c, d). The speed of clot retraction was lower in SMS2 –/– platelets than in WT platelets (Fig. 2e, f).

Next, we attempted to use the SMS2-specific inhibitor, D609 to neutralize the regulatory effects of SMS2. In the aggregation experiment, we found that D609 reduced thrombin- and collagen-induced human platelet aggregation in a dose-dependent manner. Specifically, D609 started to show an inhibitory effect on aggregation at a concentration of 10 µM. The aggregation rate was further reduced by an increase in concentration to 30 µM; when the concentration was increased to 100 µM, thrombin- and collagen-induced platelet aggregation were almost completely blocked (Fig. 2g, h). Similarly, D609 inhibited ADP- and U46619-induced human platelet aggregation in a dose-dependent manner (Supplementary Fig. 2C, D). To further test the specificity of D609, we performed platelet aggregation experiments with WT and SMS2 –/– platelets in the presence or absence of D609. D609 inhibited platelet aggregation induced by ADP, U46619, thrombin and collagen in WT platelets, but not in SMS2 –/– platelets (Supplementary Fig. 3A, B). Platelet spreading was significantly inhibited by D609 at a concentration of 100 µM (Fig. 2i, j). While low-dose D609 (10 µM) had no significant effect on clot retraction, high-dose D609 (100 µM) remarkably suppressed clot retraction (Fig. 2k, l). Our results indicate that SMS2 plays an important role in platelet activation and in outside-in signaling transduction. In addition, SMS2 might be a new target for anti-platelet treatment, considering the profound inhibitory effects of D609.

### Weakening of thrombus formation in vivo due to SMS2 deficiency

**Fig. 3:**
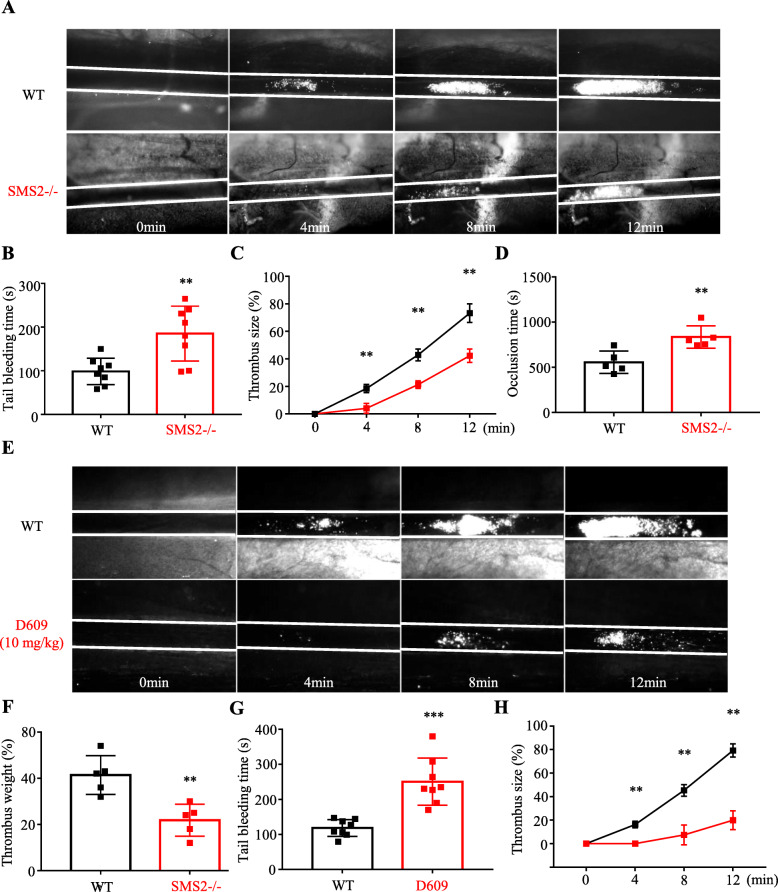
SMS2 deficiency decelerated arteriolar thrombosis. **a** Representative images of thrombus formation in mesenteric arterioles induced by ferric trichloride injury in WT and SMS2 –/– mice (upper row, WT, lower row, SMS2 –/–). Thrombus size and occlusion time were analyzed as (**c**) and (**d**) showed (*n* = 6, ** *P* < 0.01). **b** Tail bleeding time of WT and SMS2 –/– mice (*n* = 8, ** *P* < 0.01). **e** Representative images of thrombus formation in mice with or without D609 (10 mg/kg) treatment. Statistic result of thrombus size was showed in (**h**) (*n* = 6, ** *P* < 0.01). **f** Arteriovenous shunt thrombosis experiment showed D609 treatment diminished thrombus weight (*n* = 5, ** *P* < 0.01). **g** D609 prolonged tail bleeding time (*n* = 8, *** *P* < 0.001)

To verify whether SMS2 deficiency attenuated thrombus formation, we established a FeCl_3_-induced mesenteric arteriole thrombosis model. After FeCl_3_ injury, SMS2 –/– mice displayed delayed thrombus formation and arteriolar occlusion compared to WT mice (Fig. 3a, c, d). Inhibition of arteriolar thrombosis was even more prominent when WT mice were treated with D609 (10 mg/kg). After 12 min of observation, the thrombus of the D609 treatment group failed to completely occlude the mesenteric arteriole (Fig. 3e, h). In addition, through the tail bleeding test in WT and SMS2 –/– mice and the arteriovenous shunt thrombosis experiment in rats, we found that SMS2 reduction significantly prolonged bleeding time and decreased clot weight (Fig. 3b, f, g). Consistent with the impairment of platelet aggregation, spreading, and clot retraction, our results clearly indicate that SMS2 plays an important role in thrombosis and hemostasis.

### Downregulation of PLCγ/PI3K/Akt phosphorylation due to SMS2 deficiency

**Fig. 4:**
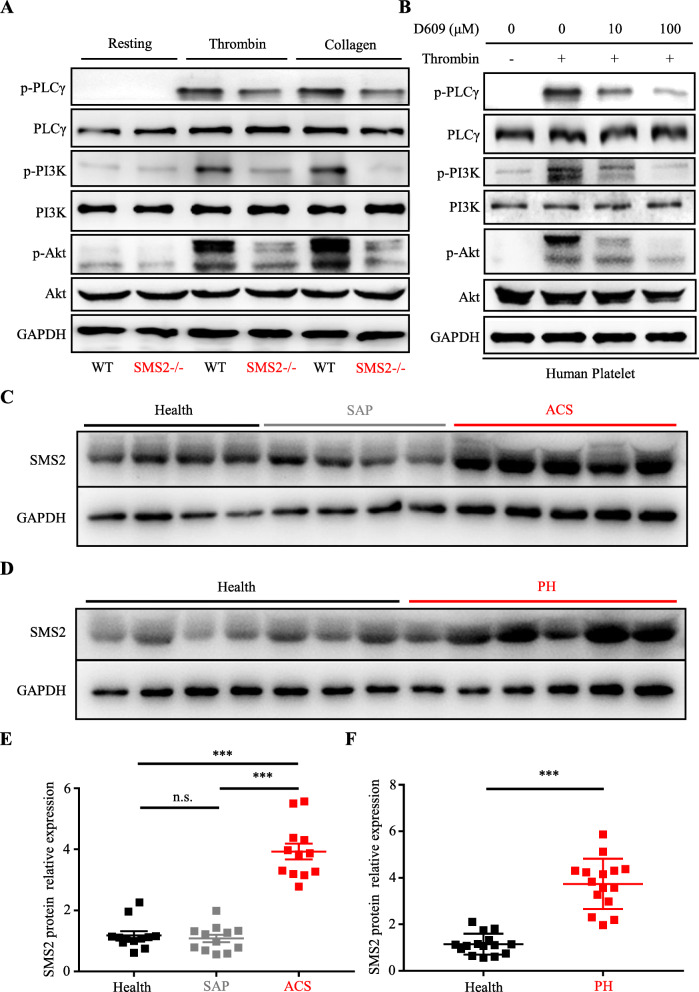
PLCγ/PI3K/Akt phosphorylation mediated reduction effects of SMS2 deficiency on platelet activation. **a** SMS2 –/– diminished thrombin- and collagen-induced PLCγ/PI3K/Akt phosphorylation. Results shown are representative images of at least 3 experiments using WT and SMS2 –/– platelets. **b** D609 weakened PLCγ/PI3K/Akt phosphorylation in human platelets after stimulated by thrombin (0.03 U/mL) in a dose-dependent manner. Results shown are representative images of at least 3 experiments. **c**-**f** SMS2 expression in healthy people and patients with SAP, ACS and PH (Health = 27, SAP = 12, ACS = 12, PH = 15, n.s. *P* > 0.05, *** *P* < 0.001)

Previous studies have suggested that SMS2 is associated with PLCγ/PI3K/Akt signaling pathway [29, 30]. To further understand the underlying mechanisms of SMS2 in the regulation of platelet activation, phosphorylation of PLCγ/PI3K/Akt in WT and SMS2 –/– platelets under the stimulation of thrombin (0.03 U/mL) and collagen (0.8 µg/mL) was investigated by immunoblotting. Our results showed that after stimulation, phosphorylation of PLCγ/PI3K/Akt was lower in SMS2 –/– platelets compared to that in the WT control group (Fig. 4a). Furthermore, phosphorylation of PLCγ/PI3K/Akt in human platelets was significantly reduced by D609 treatment (Fig. 4b).

Since the loss of SMS2 showed inhibitory effects on platelet activation and thrombosis, we assumed that patients with ACS and PH were inclined to have higher SMS2 expression compared with healthy people. Thus, we measured SMS2 expression in platelets from patients with SAP, ACS, and PH; and healthy participants. Immunoblotting results showed that SMS2 expression in patients with SAP was not significantly different compared to that in healthy subjects. On the other hand, SMS2 expression was remarkably increased in patients with ACS and PH (Fig. 4c-f). To test whether there is an association between SMS2 overexpression and platelet hyperactivity, we collected additional 19 blood samples from patients with ACS, and detected the SMS2 level and P-selectin (CD62P) exposure on platelet surface membrane with flow cytometry. We found that there is a positive correlation between SMS2 level and 0.05 U/mL thrombin-induced P-selectin exposure, indicating that increased expression level of SMS2 might associate with platelet hyperactivity (Supplementary Fig. 4A, B). Our results indicate that SMS2 is a risk factor for ACS and PH. Moreover, SMS2 provides a novel theoretical basis for anti-thrombotic treatment.

## Discussion

SM is one of the major lipids in the cell membrane, and plays a central role in the regulation of various transmembrane signaling pathways. Prior studies have shown that SM participates in lipid metabolism and may be an independent risk factor for coronary heart disease [31, 32]. As the key synthetic enzyme of SM, SMS2 –/– has been reported to decrease the development of atherosclerosis and liver steatosis in mice; the protective effect might be due to the regulation of the function of lipid microdomains on the plasma membrane [17–19]. Clinically, the continuous progression of atherosclerosis and liver steatosis could lead to ACS and PH, both of which share the common pathological process of arterial thrombosis [2, 9, 33, 34]. However, the direct influence of SMS2 deficiency on platelet activation and thrombus formation, and its association with ACS and PH, remain unclear. In this study, we identified the expression of SMS2 in mouse, rat, and human platelets; and demonstrated the crucial role of SMS2 in maintaining platelet activation and thrombosis. Loss of SMS2 leads to reduced platelet aggregation, spreading, and clot retraction, and phosphorylation of the PLCγ/PI3K/Akt signaling pathway during platelet activation is inhibited accordingly. These findings provide a novel insight into SMS2 function, indicating that SMS2 might be a mediator of hemostasis and thrombosis, as well as a therapeutic target for anti-platelet treatment. Moreover, similar outcomes were obtained when the SMS2-specific inhibitor D609 was administered. These findings provide a theoretical foundation for the use of D609 as a potential anti-platelet drug in the treatment of thrombotic diseases.

It is widely acknowledged that platelet over-activation plays a vital role in coronary artery thrombosis. Consequently, dual anti-platelet therapy consisting of aspirin and a P2Y_12_ antagonist, forms the basis of the treatment of ACS. However, a considerable number of patients with ACS still have high platelet activity and poor clinical outcomes despite the use of standard dual therapy [35]. It is therefore, required to search for novel therapeutic targets in cases of aspirin and P2Y_12_ antagonist resistance. Based on our results, patients with ACS and PH were more likely to have higher SMS2 expression than healthy individuals. Since we have demonstrated a weakening of platelet activation and thrombosis after SMS2 reduction, it is natural to assume that SMS2 is a potential therapeutic target. Furthermore, SMS2 inhibition might lead to an additional reduction in adverse events in patients with thrombotic diseases on top of regular anti-platelet treatment.

Nonetheless, our study has certain limitations. The SMS2 –/– mice used in this study were non-specific knockout mice. Since SMS2 is generally expressed in a variety of adherent cells and has various biological effects [36, 37], we cannot exclude the potential influence of other SMS2 –/– cells that might lead to the same phenotype. Further studies are required to fully understand the mechanisms underlying the regulation of platelet activation by SMS2. Although we have demonstrated that SMS2 deficiency weakens platelet activation and thrombus formation, and have found higher platelet SMS2 expression in patients with ACS and PH than in healthy subjects, it is uncertain whether SMS2 over-expression could induce platelet hyper-activation. Further research could focus on the causality between SMS2 upregulation and platelet over-activation, as well as on the morbidity of related thrombotic diseases.

## Conclusions

Our study suggests that SMS2 is an essential regulator of platelet activation and thrombus formation. SMS2 inhibitor might be a novel pharmaceutical agent in thrombotic diseases such as ACS and PH.

## Supplementary Information

Additional file 1 **Supplementary Fig. 1.** SMS2 deficiency impaired integrin αIIbβ3 activation. (A) PCR detection of SMS2 expression in platelets from WT mice, SMS2 –/– mice, SH-SY5Y cell line, rats and human. (B-E) Flow cytometry analysis of CD41 (B, C) and CD61 (D, E) expression on WT and SMS2 –/– platelets surface membrane. Resting and thrombin (0.05 U/mL) stimulated platelets were incubated with specific fluorescent antibody and detected with a flow cytometer (BD FACSAria, IIF0893488) (*n* = 5, n.s. *P* > 0.05). (F-I) Jon/A and fibrinogen binding either at resting state or under the stimulation of thrombin (0.05 U/mL) with WT and SMS2 –/– platelets (*n* = 5, n.s. *P* > 0.05, * *P* < 0.05). **Supplementary Fig. 2.** SMS2 deficiency diminished ADP- and U46619-induced platelet aggregation. (A, B) WT and SMS2 –/– platelets were stimulated with ADP and U46619 at their indicated concentrations in a platelet aggregator at 1000 rpm for 37 ℃ (n = 4, n.s. P > 0.05, * P < 0.05, ** P < 0.01). (C, D) Aggregation of human platelets stimulated by ADP (10 µM) and U46619 (0.75 µM) in the presence of various concentrations of D609 (red curve, 100 µM, purple curve, 30 µM, gray curve, 10 µM, black curve, DMSO control, n = 4, * P < 0.05, ** P < 0.01, *** P < 0.001). **Supplementary Fig. 3.** The specificity of D609 on SMS2. (A) Aggregation of WT and SMS2 –/– platelets induced by ADP (10 µM), U46619 (0.75 µM), thrombin (0.075 U/mL), and collagen (1.2 µg/mL) in the presence or absence of D609 (10 µM). Results showed that D609 inhibited platelet aggregation in WT platelets, but had no significant effect on SMS2 –/– platelets. (B) Statistic results of platelet aggregation induced by indicated agonists (n = 4, n.s. P > 0.05, * P < 0.05, ** P < 0.01). **Supplementary Fig. 4.** (A) Representative images of SMS2 level and corresponding 0.05 U/mL thrombin-induced P-selectin exposure on platelet surface membrane from patients with ACS (black curve, SMS2 low expression, red curve, SMS2 high expression). (B) A positive correlation was found between SMS2 level and corresponding P-selectin exposure (n = 19, Pearson r = 0.5826, P [two-tailed] < 0.01, GraphPad Prism version 7).

## Data Availability

All data generated or analyzed during this study are included in this published article.

## Abbreviations

SM: Sphingomyelin
SMS2: Sphingomyelin synthase 2
ACS: Acute coronary syndrome
PH: Portal hypertension
WT: Wild-type
SAP: Stable angina pectoris
PBMC: Peripheral blood mononuclear cell
RIPA: Radioimmunoprecipitation assay
PVDF: Polyvinylidene fluoride
PCR: Polymerase chain reaction
TEM: Transmission Electron Microscopy
MPV: Mean platelet volume
MFI: Mean fluorescence intensity
