# Supplementary figures and images for: The role of Sphingomyelin synthase 2 (SMS2) in platelet activation and its clinical significance
Source: Thromb J. 2021 Apr 28;19:27. doi: 10.1186/s12959-021-00282-x (PMC8082820; doi:10.1186/s12959-021-00282-x)

**A**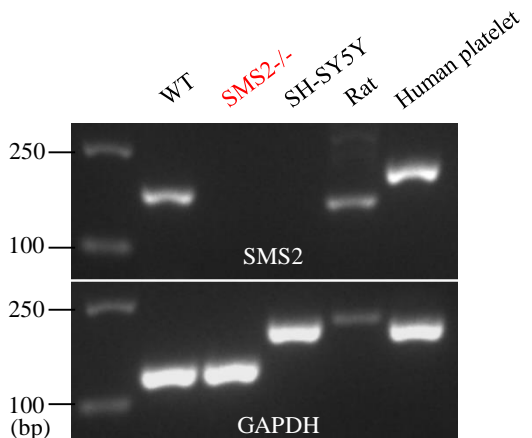**B**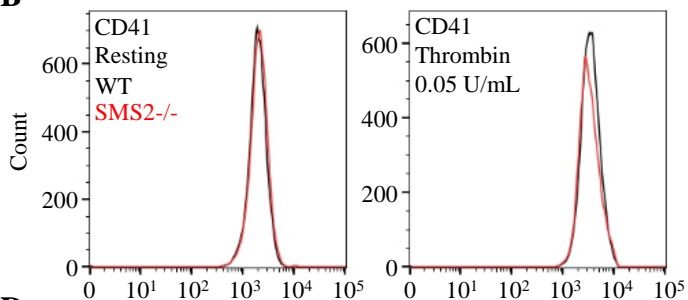**D**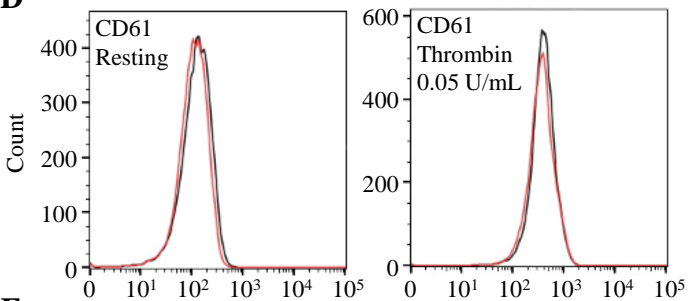**F**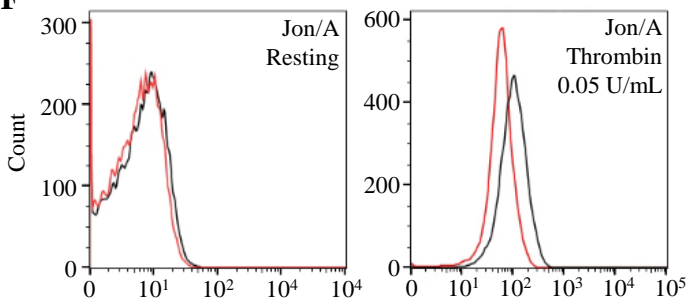**H**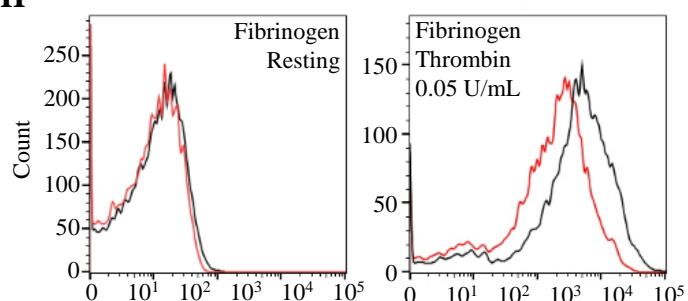

## Supplementary Figure 1

**C**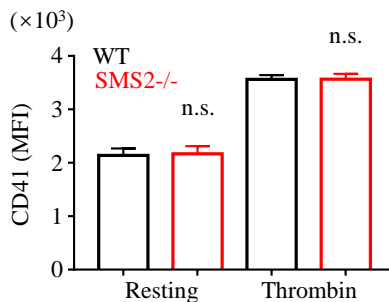**E**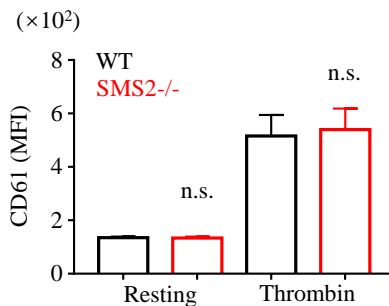**G**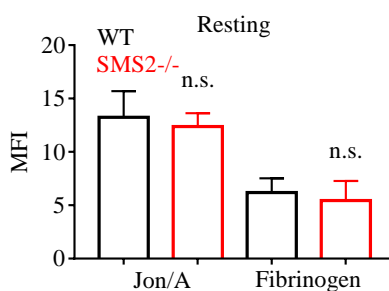**I**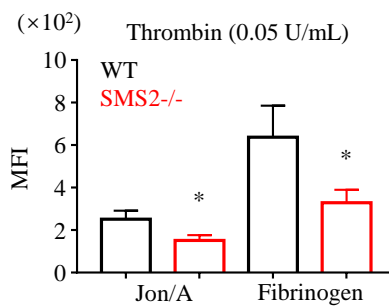

**A**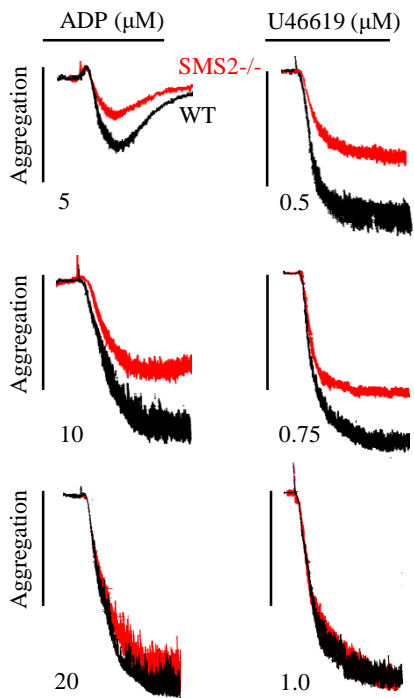**B**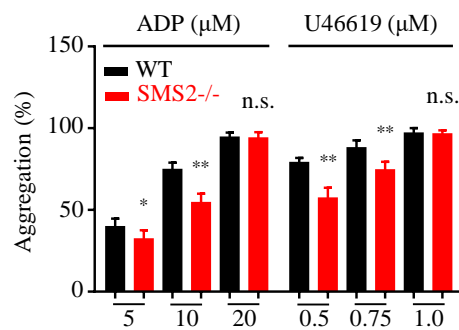**D**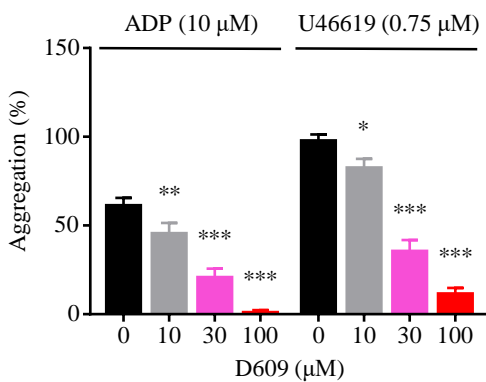**C**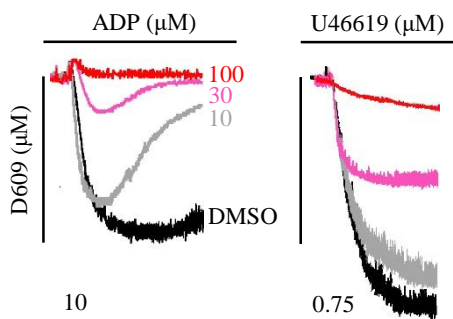

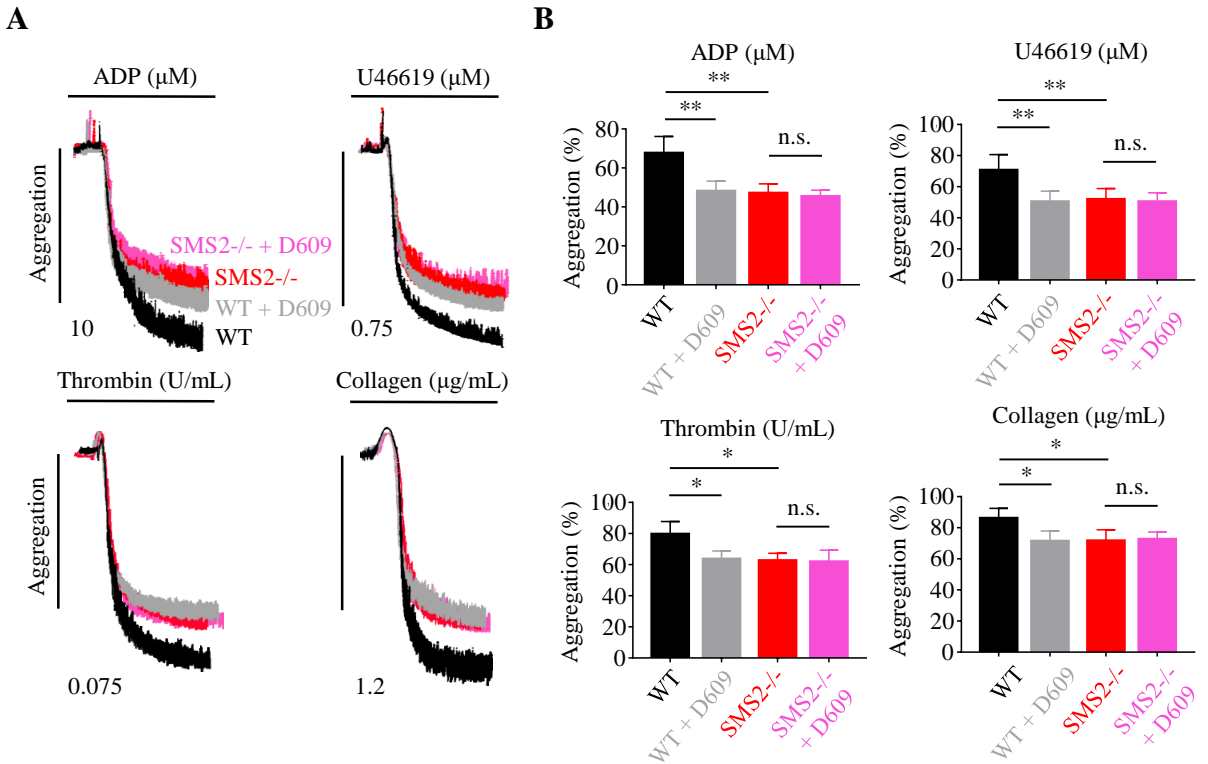

*Supplementary Figure 3*

**A**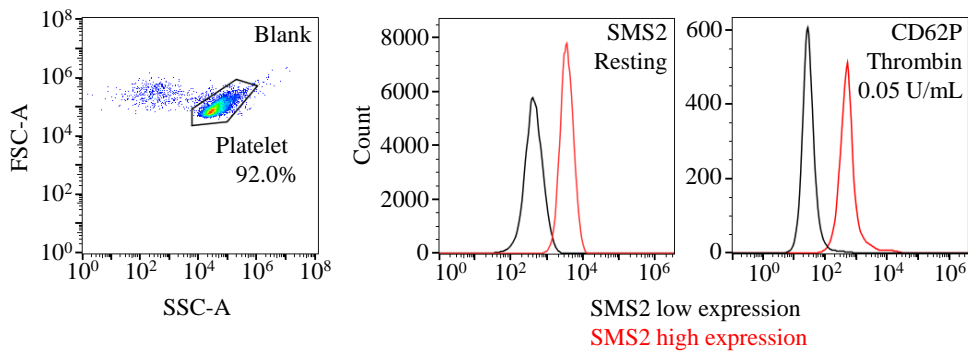**B**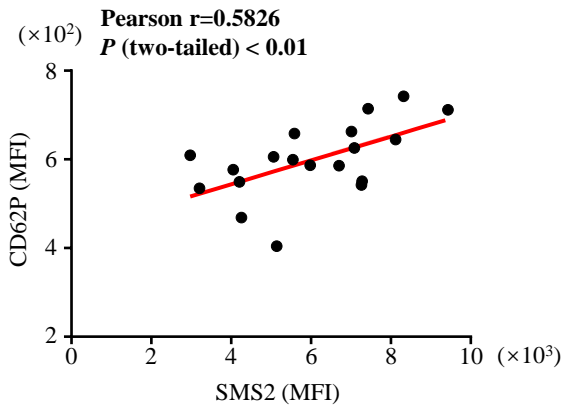

Supplement: Supplementary file 1 — Supplementary Fig. 1. SMS2 deficiency impaired integrin αIIbβ3 activation. (A) PCR detection of SMS2 expression in platelets from WT mice, SMS2 –/– mice, SH-SY5Y cell line, rats and human. (B-E) Flow cytometry analysis of CD41 (B, C) and CD61 (D, E) expression on WT and SMS2 –/– platelets surface membrane. Resting and thrombin (0.05 U/mL) stimulated platelets were incubated with specific fluorescent antibody and detected with a flow cytometer (BD FACSAria, IIF0893488) (n = 5, n.s. P > 0.05). (F-I) Jon/A and fibrinogen binding either at resting state or under the stimulation of thrombin (0.05 U/mL) with WT and SMS2 –/– platelets (n = 5, n.s. P > 0.05, * P < 0.05). Supplementary Fig. 2. SMS2 deficiency diminished ADP- and U46619-induced platelet aggregation. (A, B) WT and SMS2 –/– platelets were stimulated with ADP and U46619 at their indicated concentrations in a platelet aggregator at 1000 rpm for 37 ℃ (n = 4, n.s. P > 0.05, * P < 0.05, ** P < 0.01). (C, D) Aggregation of human platelets stimulated by ADP (10 µM) and U46619 (0.75 µM) in the presence of various concentrations of D609 (red curve, 100 µM, purple curve, 30 µM, gray curve, 10 µM, black curve, DMSO control, n = 4, * P < 0.05, ** P < 0.01, *** P < 0.001). Supplementary Fig. 3. The specificity of D609 on SMS2. (A) Aggregation of WT and SMS2 –/– platelets induced by ADP (10 µM), U46619 (0.75 µM), thrombin (0.075 U/mL), and collagen (1.2 µg/mL) in the presence or absence of D609 (10 µM). Results showed that D609 inhibited platelet aggregation in WT platelets, but had no significant effect on SMS2 –/– platelets. (B) Statistic results of platelet aggregation induced by indicated agonists (n = 4, n.s. P > 0.05, * P < 0.05, ** P < 0.01). Supplementary Fig. 4. (A) Representative images of SMS2 level and corresponding 0.05 U/mL thrombin-induced P-selectin exposure on platelet surface membrane from patients with ACS (black curve, SMS2 low expression, red curve, SMS2 high expression). (B) A positive correlation was f [file 12959_2021_282_MOESM1_ESM.pdf]
